# 2-{[3-Methyl-4-(2,2,2-trifluoro­eth­oxy)pyridin-2-yl]methyl­sulfan­yl}-1*H*-benzimidazole monohydrate

**DOI:** 10.1107/S1600536810053730

**Published:** 2011-01-08

**Authors:** Guo-Bin Ren, Ming-Huang Hong, Jia-Liang Zhong, Dong-Xu Yi, Le-Hui Xu

**Affiliations:** a Pharmaceutical Crystal Engineering Research Group, Shanghai Institute of Pharmaceutical Industry, 1320 Beijing Road (West), Shanghai 200040, People’s Republic of China

## Abstract

The asymmetric unit of the title compound, C_16_H_14_F_3_N_3_OS·H_2_O, contains two independent mol­ecules (*A* and *B*) and two water mol­ecules, one of which is disordered over two positions in a 0.790 (8):0.210 (8) ratio. The mol­ecular conformations are close, the benzimidazole mean plane and pyridine ring forming dihedral angles of 1.8 (3) and 0.1 (2)° in mol­ecules *A* and *B*, respectively. The water mol­ecules are involved in formation of two independent hydrogen-bonded chains *via* N—H⋯O and O—H⋯N hydrogen bonds. Chains propagating along the *a* axis are formed by mol­ecule *A* and one independent water mol­ecule, while chains propagating along the *b* axis are formed by mol­ecule *B* and the other independent water mol­ecule. The crystal packing exhibits π–π inter­actions, as indicated by short distances of 3.607 (3) and 3.701 (3) Å between the centroids of the imidazole and pyridine rings of neighbouring mol­ecules.

## Related literature

The title compound is an inter­mediate in the synthesis of the anti-ulcer drug lansoprazole [systematic name (*R*S)-2-([3-methyl-4-(2,2,2-trifluoro­eth­oxy)pyridin-2-yl]methyl­sulfin­yl)-1*H*-benzo[*d*]imidazole], see: Del Rio *et al.* (2007 ▶); Reddy *et al.* (2008 ▶); Iwahi *et al.* (1991 ▶). For related structures, see: Swamy & Ravikumar (2007 ▶); Hakim Al-arique *et al.* (2010 ▶).
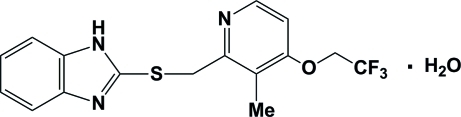


## Experimental

### 

#### Crystal data

C_16_H_14_F_3_N_3_OS·H_2_O
*M* *_r_* = 371.39
Triclinic, 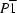

*a* = 7.3526 (1) Å
*b* = 7.4702 (1) Å
*c* = 30.6500 (3) Å
α = 88.27°
β = 87.79°
γ = 89.13°
*V* = 1681.27 (4) Å^3^
*Z* = 4
Cu *K*α radiation
μ = 2.15 mm^−1^
*T* = 296 K
0.28 × 0.12 × 0.10 mm

#### Data collection

Bruker SMART APEX diffractometer
12461 measured reflections
5446 independent reflections
5282 reflections with *I* > 2σ(*I*)
*R*_int_ = 0.017

#### Refinement

*R*[*F* ^2^ > 2σ(*F* ^2^)] = 0.041
*wR*(*F* ^2^) = 0.117
*S* = 1.06
5446 reflections
462 parameters
H-atom parameters constrained
Δρ_max_ = 0.41 e Å^−3^
Δρ_min_ = −0.32 e Å^−3^

### 

Data collection: *SMART* (Bruker, 2005 ▶); cell refinement: *SAINT* (Bruker, 2005 ▶); data reduction: *SAINT*; program(s) used to solve structure: *SHELXS97* (Sheldrick, 2008 ▶); program(s) used to refine structure: *SHELXL97* (Sheldrick, 2008 ▶); molecular graphics: *XP* in *SHELXTL* (Sheldrick, 2008 ▶); software used to prepare material for publication: *SHELXL97*.

## Supplementary Material

Crystal structure: contains datablocks I, global. DOI: 10.1107/S1600536810053730/cv5015sup1.cif

Structure factors: contains datablocks I. DOI: 10.1107/S1600536810053730/cv5015Isup2.hkl

Additional supplementary materials:  crystallographic information; 3D view; checkCIF report

## Figures and Tables

**Table 1:** Hydrogen-bond geometry (Å, °)

*D*—H⋯*A*	*D*—H	H⋯*A*	*D*⋯*A*	*D*—H⋯*A*
N2*A*—H2*AA*⋯O*A*	0.86	1.95	2.771 (2)	161
O*A*—H*A*1⋯N1*A*^i^	0.83	2.00	2.806 (2)	161
N2*B*—H2*BA*⋯O*B*	0.86	1.98	2.799 (3)	160
O*B*—H*B*1⋯N1*B*^ii^	0.84	2.03	2.798 (3)	152

Symmetry codes: (i) 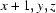; (ii) 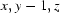.

**Fig. 1.**
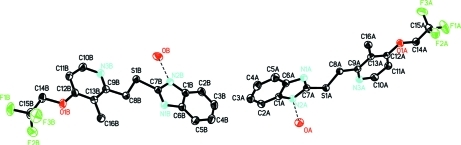
The content of asymmetric unit (I) showing the atom-labelling scheme. Displacement ellipsoids are drawn at the 30% probability level. Dashed lines denote hydrogen bonds. Only major component of the disordered water molecule is shown. H atoms have been omitted for clarity.

**Fig. 2.**
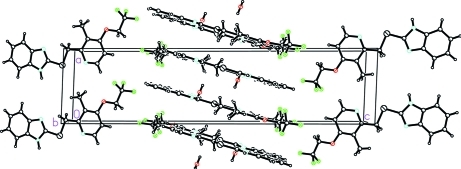
A packing diagram.

## supplementary crystallographic information

### Comment

The title compound, (I), is important intermediate in the synthesis of lansoprazole (Del Rio *et al.*, 2007; Reddy *et al.*, 2008), which exhibits anti-ulcer effect (Iwahi *et al.*,1991). Herewith we present its crystal structure.

The asymmetric unit of (I) contains two independent molecules (Fig. 1), A and B, respectively, and two crystalline water molecules, one of which is disordered over two positions in a ratio 0.790 (8):0.210 (8). The molecular conformations of A and B are close - the benzimidazole mean plane and pyridine ring form the dihedral angles of 1.8 (3)° and 0.1 (2)° in A and B, respectively. The bond lengths and angles in A and B are normal and comparable with those observed in the related compounds (Swamy *et al.*, 2007; Hakim,*et al.*, 2010). The torsion angle of C7—S1—C8—C9 in A is 178.85 (12) ° (179.88 (14) ° in B).

The crystalline water molecules are involved in formation of two independent hydrogen-bonded chains *via* N—H···O and O—H···N hydrogen bonds (Table 1). The chains propagating along the axis *a* are formed by the molecule A and one independent water molecule, while the chains propagating along the axis *b* are formed by the molecule B and another independent water molecule. The crystal packing exhibits π-π interactions proved by short distances of 3.607 (3) and 3.701 (3) Å between the centroids of imidazole and pyridine rings from the neighbouring molecules.

### Experimental

The raw material was kindly provided by Shanghai Enran Sci-Tech Investment Management Co., Ltd.The compound was dissolved in acetonitrile and suitable crystals of X-ray were obtained by slow evaporation at room temperature over a period of one week.

### Refinement

Water H atoms were initially located in a difference Fourier map (O—H  0.80-0.85 Å), and refined as riding, with *U*_iso_(H) = 1.5 *U*_eq_(O). All other H atoms were constrained to an ideal geometry (C—H 0.93 - 0.97 Å; N—H 0.86 Å). All H atoms were refined as riding, with and *U*_iso_(H) = 1.2 - 1.5 *U*_eq_ of the parent atom. One water molecule (OB) has been treated as disordered between two positions with the occupancies refined to 0.790 (8) and 0.210 (8), respectively.

### Crystal data

**Table:**

C_16_H_14_F_3_N_3_OS·H_2_O	*Z* = 4
*M**_r_* = 371.39	*F*(000) = 768
Triclinic, *P*1	*D*_x_ = 1.467 Mg m^−^^3^
Hall symbol: -P 1	Cu *K*α radiation, λ = 1.54178 Å
*a* = 7.3526 (1) Å	Cell parameters from 9679 reflections
*b* = 7.4702 (1) Å	θ = 5.8–67.1°
*c* = 30.6500 (3) Å	µ = 2.15 mm^−^^1^
α = 88.27°	*T* = 296 K
β = 87.79°	Prism, colourless
γ = 89.13°	0.28 × 0.12 × 0.10 mm
*V* = 1681.27 (4) Å^3^	

### Data collection

**Table:**

Bruker SMART APEX diffractometer	5282 reflections with *I* > 2σ(*I*)
Radiation source: sealed tube	*R*_int_ = 0.017
graphite	θ_max_ = 67.4°, θ_min_ = 4.3°
φ and ω scans	*h* = −7→8
12461 measured reflections	*k* = −8→8
5446 independent reflections	*l* = −33→36

### Refinement

**Table:**

Refinement on *F*^2^	Secondary atom site location: difference Fourier map
Least-squares matrix: full	Hydrogen site location: inferred from neighbouring sites
*R*[*F*^2^ > 2σ(*F*^2^)] = 0.041	H-atom parameters constrained
*wR*(*F*^2^) = 0.117	*w* = 1/[σ^2^(*F*_o_^2^) + (0.0671*P*)^2^ + 0.6061*P*] where *P* = (*F*_o_^2^ + 2*F*_c_^2^)/3
*S* = 1.06	(Δ/σ)_max_ = 0.001
5446 reflections	Δρ_max_ = 0.41 e Å^−^^3^
462 parameters	Δρ_min_ = −0.32 e Å^−^^3^
0 restraints	Extinction correction: *SHELXL97* (Sheldrick, 2008), Fc^*^=kFc[1+0.001xFc^2^λ^3^/sin(2θ)]^-1/4^
Primary atom site location: structure-invariant direct methods	Extinction coefficient: 0.0062 (5)

### Special details

**Table:**

Geometry. All e.s.d.'s (except the e.s.d. in the dihedral angle between two l.s. planes) are estimated using the full covariance matrix. The cell e.s.d.'s are taken into account individually in the estimation of e.s.d.'s in distances, angles and torsion angles; correlations between e.s.d.'s in cell parameters are only used when they are defined by crystal symmetry. An approximate (isotropic) treatment of cell e.s.d.'s is used for estimating e.s.d.'s involving l.s. planes.
Refinement. Refinement of *F*^2^ against ALL reflections. The weighted *R*-factor *wR* and goodness of fit *S* are based on *F*^2^, conventional *R*-factors *R* are based on *F*, with *F* set to zero for negative *F*^2^. The threshold expression of *F*^2^ > σ(*F*^2^) is used only for calculating *R*-factors(gt) *etc*. and is not relevant to the choice of reflections for refinement. *R*-factors based on *F*^2^ are statistically about twice as large as those based on *F*, and *R*- factors based on ALL data will be even larger.

### Fractional atomic coordinates and isotropic or equivalent isotropic displacement parameters (Å^2^)

**Table:**

	*x*	*y*	*z*	*U*_iso_*/*U*_eq_	Occ. (<1)
S1A	0.20282 (6)	0.80754 (6)	0.034292 (14)	0.04220 (15)	
F1A	−0.4248 (3)	0.4400 (3)	−0.22095 (6)	0.1133 (7)	
F2A	−0.5441 (2)	0.6396 (2)	−0.18024 (5)	0.0767 (4)	
F3A	−0.5417 (2)	0.3690 (2)	−0.15787 (6)	0.0898 (5)	
O1A	−0.28512 (19)	0.57155 (19)	−0.11848 (4)	0.0499 (3)	
N1A	−0.0279 (2)	0.8860 (2)	0.10435 (5)	0.0449 (4)	
N2A	0.2693 (2)	0.9203 (2)	0.11406 (5)	0.0419 (3)	
H2AA	0.3847	0.9196	0.1083	0.050*	
N3A	0.1655 (2)	0.6976 (2)	−0.04904 (5)	0.0480 (4)	
C1A	0.1826 (3)	0.9679 (2)	0.15275 (6)	0.0414 (4)	
C2A	0.2464 (3)	1.0248 (3)	0.19196 (7)	0.0516 (5)	
H2AB	0.3702	1.0347	0.1964	0.062*	
C3A	0.1170 (3)	1.0661 (3)	0.22414 (7)	0.0593 (6)	
H3AA	0.1546	1.1059	0.2508	0.071*	
C4A	−0.0675 (3)	1.0495 (3)	0.21770 (7)	0.0615 (6)	
H4AA	−0.1507	1.0806	0.2399	0.074*	
C5A	−0.1308 (3)	0.9882 (3)	0.17920 (7)	0.0568 (5)	
H5AA	−0.2547	0.9751	0.1754	0.068*	
C6A	−0.0026 (3)	0.9463 (2)	0.14626 (6)	0.0432 (4)	
C7A	0.1375 (2)	0.8745 (2)	0.08674 (6)	0.0390 (4)	
C8A	−0.0216 (2)	0.7654 (2)	0.01491 (6)	0.0407 (4)	
H8AA	−0.0798	0.6726	0.0331	0.049*	
H8AB	−0.0961	0.8734	0.0167	0.049*	
C9A	−0.0052 (2)	0.7070 (2)	−0.03177 (6)	0.0384 (4)	
C10A	0.1846 (3)	0.6412 (3)	−0.08980 (7)	0.0543 (5)	
H10A	0.3019	0.6321	−0.1021	0.065*	
C11A	0.0431 (3)	0.5958 (3)	−0.11481 (6)	0.0504 (5)	
H11A	0.0638	0.5563	−0.1431	0.061*	
C12A	−0.1325 (3)	0.6103 (2)	−0.09671 (6)	0.0415 (4)	
C13A	−0.1595 (2)	0.6663 (2)	−0.05382 (6)	0.0388 (4)	
C14A	−0.2595 (3)	0.5145 (3)	−0.16192 (6)	0.0495 (5)	
H14A	−0.1914	0.4023	−0.1624	0.059*	
H14B	−0.1921	0.6033	−0.1794	0.059*	
C15A	−0.4423 (4)	0.4908 (3)	−0.17966 (7)	0.0618 (6)	
C16A	−0.3470 (3)	0.6790 (3)	−0.03280 (7)	0.0491 (5)	
H16A	−0.3389	0.7198	−0.0035	0.074*	
H16B	−0.4021	0.5632	−0.0321	0.074*	
H16C	−0.4198	0.7621	−0.0494	0.074*	
OA	0.6171 (2)	0.9501 (2)	0.07662 (6)	0.0674 (4)	
HA1	0.7128	0.9071	0.0867	0.101*	
HA2	0.6392	1.0447	0.0630	0.101*	
S1B	0.26211 (7)	0.81787 (6)	0.488240 (15)	0.04885 (16)	
F1B	0.0052 (3)	1.4472 (3)	0.75578 (5)	0.1092 (7)	
F2B	0.1852 (3)	1.5488 (2)	0.70486 (6)	0.0992 (6)	
F3B	−0.0976 (3)	1.5730 (2)	0.69808 (6)	0.1053 (6)	
O1B	0.0650 (2)	1.30722 (19)	0.64771 (4)	0.0576 (4)	
N1B	0.3484 (2)	1.0373 (2)	0.41683 (5)	0.0485 (4)	
N2B	0.3662 (3)	0.7438 (2)	0.40584 (5)	0.0506 (4)	
H2BA	0.3605	0.6305	0.4114	0.061*	
N3B	0.1553 (3)	0.8612 (2)	0.57285 (6)	0.0578 (5)	
C1B	0.4148 (3)	0.8255 (3)	0.36638 (6)	0.0479 (4)	
C2B	0.4657 (3)	0.7591 (3)	0.32581 (7)	0.0604 (6)	
H2BB	0.4733	0.6367	0.3212	0.072*	
C3B	0.5043 (3)	0.8839 (4)	0.29259 (7)	0.0652 (6)	
H3BA	0.5386	0.8444	0.2649	0.078*	
C4B	0.4932 (3)	1.0663 (4)	0.29952 (7)	0.0648 (6)	
H4BA	0.5205	1.1463	0.2765	0.078*	
C5B	0.4428 (3)	1.1314 (3)	0.33978 (7)	0.0605 (6)	
H5BA	0.4354	1.2540	0.3442	0.073*	
C6B	0.4032 (3)	1.0091 (3)	0.37361 (6)	0.0480 (4)	
C7B	0.3289 (3)	0.8765 (3)	0.43438 (6)	0.0443 (4)	
C8B	0.2354 (3)	1.0420 (3)	0.50903 (6)	0.0473 (4)	
H8BA	0.3498	1.1047	0.5058	0.057*	
H8BB	0.1450	1.1088	0.4927	0.057*	
C9B	0.1760 (3)	1.0277 (3)	0.55651 (6)	0.0461 (4)	
C10B	0.1024 (4)	0.8433 (3)	0.61464 (7)	0.0637 (6)	
H10B	0.0867	0.7280	0.6263	0.076*	
C11B	0.0695 (3)	0.9845 (3)	0.64178 (7)	0.0562 (5)	
H11B	0.0327	0.9657	0.6709	0.067*	
C12B	0.0929 (3)	1.1550 (3)	0.62422 (6)	0.0479 (4)	
C13B	0.1465 (3)	1.1813 (3)	0.58049 (6)	0.0458 (4)	
C14B	0.0293 (3)	1.2837 (3)	0.69304 (6)	0.0558 (5)	
H14C	0.1216	1.2065	0.7058	0.067*	
H14D	−0.0884	1.2288	0.6986	0.067*	
C15B	0.0308 (4)	1.4636 (4)	0.71260 (8)	0.0706 (7)	
C16B	0.1682 (4)	1.3666 (3)	0.56065 (7)	0.0620 (6)	
H16D	0.1412	1.4535	0.5825	0.093*	
H16E	0.0861	1.3836	0.5372	0.093*	
H16F	0.2911	1.3811	0.5495	0.093*	
OB	0.4154 (6)	0.3918 (3)	0.43764 (9)	0.0886 (14)	0.790 (8)
OB'	0.251 (2)	0.3984 (11)	0.4323 (3)	0.090 (5)	0.210 (8)
HB1	0.3586	0.2971	0.4339	0.134*	0.790 (8)
HB1'	0.3361	0.3261	0.4312	0.134*	0.210 (8)
HB2	0.5139	0.3549	0.4455	0.134*	0.790 (8)
HB2'	0.1581	0.3375	0.4272	0.134*	0.210 (8)

### Atomic displacement parameters (Å^2^)

**Table:**

	*U*^11^	*U*^22^	*U*^33^	*U*^12^	*U*^13^	*U*^23^
S1A	0.0356 (3)	0.0494 (3)	0.0416 (3)	−0.00072 (17)	0.00079 (18)	−0.00341 (18)
F1A	0.0937 (13)	0.179 (2)	0.0719 (10)	0.0062 (12)	−0.0171 (9)	−0.0662 (12)
F2A	0.0676 (10)	0.0855 (10)	0.0770 (9)	0.0115 (7)	−0.0118 (7)	0.0009 (7)
F3A	0.0722 (11)	0.0777 (10)	0.1210 (14)	−0.0239 (8)	−0.0155 (9)	−0.0013 (9)
O1A	0.0464 (8)	0.0642 (8)	0.0394 (7)	−0.0068 (6)	0.0000 (6)	−0.0081 (6)
N1A	0.0361 (9)	0.0526 (9)	0.0458 (8)	−0.0014 (6)	−0.0003 (7)	−0.0009 (7)
N2A	0.0322 (8)	0.0490 (8)	0.0445 (8)	−0.0006 (6)	−0.0006 (6)	−0.0029 (6)
N3A	0.0378 (9)	0.0584 (10)	0.0476 (9)	−0.0039 (7)	0.0042 (7)	−0.0056 (7)
C1A	0.0427 (11)	0.0390 (9)	0.0423 (9)	0.0002 (7)	0.0001 (8)	0.0014 (7)
C2A	0.0512 (13)	0.0535 (11)	0.0506 (11)	−0.0028 (9)	−0.0058 (9)	−0.0042 (9)
C3A	0.0729 (16)	0.0612 (13)	0.0440 (11)	−0.0026 (10)	0.0011 (10)	−0.0059 (9)
C4A	0.0666 (16)	0.0698 (14)	0.0469 (11)	0.0042 (11)	0.0141 (10)	−0.0032 (10)
C5A	0.0449 (12)	0.0698 (14)	0.0547 (12)	0.0005 (9)	0.0086 (9)	0.0013 (10)
C6A	0.0416 (11)	0.0453 (10)	0.0424 (9)	0.0005 (7)	0.0013 (8)	0.0016 (7)
C7A	0.0359 (10)	0.0379 (9)	0.0431 (9)	0.0007 (7)	−0.0021 (7)	0.0024 (7)
C8A	0.0368 (10)	0.0440 (9)	0.0411 (9)	−0.0010 (7)	−0.0004 (7)	−0.0010 (7)
C9A	0.0375 (10)	0.0368 (9)	0.0404 (9)	−0.0013 (7)	0.0023 (7)	0.0012 (7)
C10A	0.0404 (11)	0.0708 (13)	0.0513 (11)	−0.0056 (9)	0.0109 (9)	−0.0083 (9)
C11A	0.0508 (12)	0.0587 (12)	0.0414 (10)	−0.0043 (9)	0.0089 (8)	−0.0065 (8)
C12A	0.0425 (11)	0.0407 (9)	0.0410 (9)	−0.0049 (7)	−0.0005 (8)	0.0010 (7)
C13A	0.0398 (10)	0.0361 (9)	0.0400 (9)	−0.0011 (7)	0.0036 (7)	0.0010 (7)
C14A	0.0578 (13)	0.0492 (11)	0.0416 (10)	0.0000 (8)	0.0010 (9)	−0.0073 (8)
C15A	0.0673 (15)	0.0666 (14)	0.0526 (12)	0.0008 (11)	−0.0056 (10)	−0.0176 (10)
C16A	0.0382 (11)	0.0578 (11)	0.0512 (11)	−0.0022 (8)	0.0025 (8)	−0.0053 (9)
OA	0.0362 (9)	0.0880 (12)	0.0770 (11)	−0.0009 (7)	0.0032 (7)	0.0062 (9)
S1B	0.0613 (3)	0.0435 (3)	0.0417 (3)	−0.0018 (2)	0.0023 (2)	−0.00479 (19)
F1B	0.184 (2)	0.0958 (12)	0.0464 (8)	0.0074 (12)	0.0272 (10)	−0.0194 (8)
F2B	0.1211 (15)	0.0910 (12)	0.0870 (11)	−0.0297 (10)	0.0105 (10)	−0.0319 (9)
F3B	0.1288 (16)	0.0833 (11)	0.1016 (13)	0.0405 (11)	0.0145 (11)	−0.0096 (9)
O1B	0.0808 (11)	0.0539 (8)	0.0378 (7)	0.0024 (7)	0.0050 (7)	−0.0076 (6)
N1B	0.0569 (10)	0.0439 (9)	0.0445 (8)	0.0010 (7)	0.0025 (7)	−0.0065 (7)
N2B	0.0636 (11)	0.0399 (8)	0.0485 (9)	−0.0004 (7)	0.0033 (8)	−0.0091 (7)
N3B	0.0814 (14)	0.0469 (9)	0.0449 (9)	−0.0054 (8)	0.0032 (9)	−0.0030 (7)
C1B	0.0470 (12)	0.0527 (11)	0.0443 (10)	0.0000 (8)	−0.0010 (8)	−0.0089 (8)
C2B	0.0628 (15)	0.0654 (13)	0.0539 (12)	−0.0007 (10)	0.0010 (10)	−0.0197 (10)
C3B	0.0583 (15)	0.0947 (18)	0.0430 (11)	−0.0019 (12)	0.0022 (10)	−0.0127 (11)
C4B	0.0622 (15)	0.0843 (17)	0.0470 (11)	−0.0021 (11)	0.0016 (10)	0.0063 (11)
C5B	0.0670 (15)	0.0596 (13)	0.0545 (12)	−0.0006 (10)	0.0006 (10)	0.0029 (10)
C6B	0.0473 (12)	0.0528 (11)	0.0438 (10)	0.0017 (8)	−0.0009 (8)	−0.0043 (8)
C7B	0.0458 (11)	0.0443 (10)	0.0430 (10)	−0.0001 (7)	−0.0016 (8)	−0.0080 (8)
C8B	0.0554 (12)	0.0438 (10)	0.0428 (10)	−0.0037 (8)	0.0031 (8)	−0.0070 (8)
C9B	0.0480 (12)	0.0484 (10)	0.0420 (10)	−0.0038 (8)	−0.0018 (8)	−0.0031 (8)
C10B	0.0935 (19)	0.0489 (12)	0.0483 (11)	−0.0076 (11)	0.0039 (11)	0.0012 (9)
C11B	0.0701 (15)	0.0587 (12)	0.0393 (10)	−0.0053 (10)	0.0018 (9)	0.0002 (9)
C12B	0.0508 (12)	0.0532 (11)	0.0404 (10)	−0.0020 (8)	−0.0022 (8)	−0.0087 (8)
C13B	0.0499 (12)	0.0472 (10)	0.0407 (9)	−0.0037 (8)	−0.0012 (8)	−0.0037 (8)
C14B	0.0641 (14)	0.0643 (13)	0.0386 (10)	0.0024 (10)	0.0058 (9)	−0.0062 (9)
C15B	0.092 (2)	0.0712 (15)	0.0477 (12)	0.0052 (14)	0.0155 (12)	−0.0112 (11)
C16B	0.0879 (18)	0.0469 (11)	0.0511 (12)	−0.0078 (10)	0.0046 (11)	−0.0043 (9)
OB	0.121 (4)	0.0422 (11)	0.1049 (19)	−0.0115 (12)	−0.0339 (17)	0.0035 (11)
OB'	0.119 (13)	0.046 (4)	0.103 (7)	0.015 (5)	0.006 (7)	−0.008 (4)

### Geometric parameters (Å, °)

**Table:**

S1A—C7A	1.7453 (18)	F1B—C15B	1.332 (3)
S1A—C8A	1.8107 (18)	F2B—C15B	1.320 (3)
F1A—C15A	1.333 (3)	F3B—C15B	1.321 (3)
F2A—C15A	1.331 (3)	O1B—C12B	1.373 (2)
F3A—C15A	1.324 (3)	O1B—C14B	1.410 (2)
O1A—C12A	1.367 (2)	N1B—C7B	1.309 (3)
O1A—C14A	1.415 (2)	N1B—C6B	1.391 (2)
N1A—C7A	1.314 (2)	N2B—C7B	1.360 (2)
N1A—C6A	1.394 (2)	N2B—C1B	1.375 (3)
N2A—C7A	1.359 (2)	N2B—H2BA	0.8600
N2A—C1A	1.379 (2)	N3B—C10B	1.328 (3)
N2A—H2AA	0.8600	N3B—C9B	1.335 (3)
N3A—C10A	1.332 (3)	C1B—C2B	1.388 (3)
N3A—C9A	1.344 (2)	C1B—C6B	1.397 (3)
C1A—C2A	1.387 (3)	C2B—C3B	1.384 (4)
C1A—C6A	1.396 (3)	C2B—H2BB	0.9300
C2A—C3A	1.382 (3)	C3B—C4B	1.386 (4)
C2A—H2AB	0.9300	C3B—H3BA	0.9300
C3A—C4A	1.386 (4)	C4B—C5B	1.376 (3)
C3A—H3AA	0.9300	C4B—H4BA	0.9300
C4A—C5A	1.378 (3)	C5B—C6B	1.386 (3)
C4A—H4AA	0.9300	C5B—H5BA	0.9300
C5A—C6A	1.393 (3)	C8B—C9B	1.504 (3)
C5A—H5AA	0.9300	C8B—H8BA	0.9700
C8A—C9A	1.508 (2)	C8B—H8BB	0.9700
C8A—H8AA	0.9700	C9B—C13B	1.391 (3)
C8A—H8AB	0.9700	C10B—C11B	1.376 (3)
C9A—C13A	1.385 (3)	C10B—H10B	0.9300
C10A—C11A	1.369 (3)	C11B—C12B	1.378 (3)
C10A—H10A	0.9300	C11B—H11B	0.9300
C11A—C12A	1.389 (3)	C12B—C13B	1.391 (3)
C11A—H11A	0.9300	C13B—C16B	1.504 (3)
C12A—C13A	1.398 (3)	C14B—C15B	1.488 (3)
C13A—C16A	1.502 (3)	C14B—H14C	0.9700
C14A—C15A	1.485 (3)	C14B—H14D	0.9700
C14A—H14A	0.9700	C16B—H16D	0.9600
C14A—H14B	0.9700	C16B—H16E	0.9600
C16A—H16A	0.9600	C16B—H16F	0.9600
C16A—H16B	0.9600	OB—HB1	0.8399
C16A—H16C	0.9600	OB—HB1'	0.8013
OA—HA1	0.8349	OB—HB2	0.8132
OA—HA2	0.8249	OB'—HB1	1.0873
S1B—C7B	1.7490 (19)	OB'—HB1'	0.8201
S1B—C8B	1.8146 (19)	OB'—HB2'	0.8488
			
C7A—S1A—C8A	98.11 (8)	C7B—N2B—C1B	106.89 (16)
C12A—O1A—C14A	117.06 (15)	C7B—N2B—H2BA	126.6
C7A—N1A—C6A	104.30 (16)	C1B—N2B—H2BA	126.6
C7A—N2A—C1A	106.86 (15)	C10B—N3B—C9B	117.15 (18)
C7A—N2A—H2AA	126.6	N2B—C1B—C2B	132.8 (2)
C1A—N2A—H2AA	126.6	N2B—C1B—C6B	105.28 (16)
C10A—N3A—C9A	116.71 (17)	C2B—C1B—C6B	122.0 (2)
N2A—C1A—C2A	132.64 (19)	C3B—C2B—C1B	116.8 (2)
N2A—C1A—C6A	105.19 (16)	C3B—C2B—H2BB	121.6
C2A—C1A—C6A	122.17 (18)	C1B—C2B—H2BB	121.6
C3A—C2A—C1A	116.8 (2)	C2B—C3B—C4B	121.6 (2)
C3A—C2A—H2AB	121.6	C2B—C3B—H3BA	119.2
C1A—C2A—H2AB	121.6	C4B—C3B—H3BA	119.2
C2A—C3A—C4A	121.6 (2)	C5B—C4B—C3B	121.4 (2)
C2A—C3A—H3AA	119.2	C5B—C4B—H4BA	119.3
C4A—C3A—H3AA	119.2	C3B—C4B—H4BA	119.3
C5A—C4A—C3A	121.7 (2)	C4B—C5B—C6B	118.1 (2)
C5A—C4A—H4AA	119.2	C4B—C5B—H5BA	120.9
C3A—C4A—H4AA	119.2	C6B—C5B—H5BA	120.9
C4A—C5A—C6A	117.7 (2)	C5B—C6B—N1B	130.08 (19)
C4A—C5A—H5AA	121.2	C5B—C6B—C1B	120.15 (19)
C6A—C5A—H5AA	121.2	N1B—C6B—C1B	109.77 (17)
C5A—C6A—N1A	129.8 (2)	N1B—C7B—N2B	113.33 (17)
C5A—C6A—C1A	120.08 (19)	N1B—C7B—S1B	127.96 (14)
N1A—C6A—C1A	110.09 (16)	N2B—C7B—S1B	118.72 (14)
N1A—C7A—N2A	113.55 (16)	C9B—C8B—S1B	108.66 (13)
N1A—C7A—S1A	127.99 (14)	C9B—C8B—H8BA	110.0
N2A—C7A—S1A	118.46 (13)	S1B—C8B—H8BA	110.0
C9A—C8A—S1A	109.41 (12)	C9B—C8B—H8BB	110.0
C9A—C8A—H8AA	109.8	S1B—C8B—H8BB	110.0
S1A—C8A—H8AA	109.8	H8BA—C8B—H8BB	108.3
C9A—C8A—H8AB	109.8	N3B—C9B—C13B	124.19 (18)
S1A—C8A—H8AB	109.8	N3B—C9B—C8B	115.44 (17)
H8AA—C8A—H8AB	108.2	C13B—C9B—C8B	120.37 (17)
N3A—C9A—C13A	124.41 (16)	N3B—C10B—C11B	124.2 (2)
N3A—C9A—C8A	115.40 (16)	N3B—C10B—H10B	117.9
C13A—C9A—C8A	120.19 (15)	C11B—C10B—H10B	117.9
N3A—C10A—C11A	124.36 (18)	C10B—C11B—C12B	117.57 (19)
N3A—C10A—H10A	117.8	C10B—C11B—H11B	121.2
C11A—C10A—H10A	117.8	C12B—C11B—H11B	121.2
C10A—C11A—C12A	118.03 (18)	O1B—C12B—C11B	123.44 (18)
C10A—C11A—H11A	121.0	O1B—C12B—C13B	115.97 (17)
C12A—C11A—H11A	121.0	C11B—C12B—C13B	120.59 (18)
O1A—C12A—C11A	123.68 (17)	C9B—C13B—C12B	116.33 (18)
O1A—C12A—C13A	116.54 (16)	C9B—C13B—C16B	122.53 (18)
C11A—C12A—C13A	119.78 (18)	C12B—C13B—C16B	121.12 (18)
C9A—C13A—C12A	116.69 (16)	O1B—C14B—C15B	107.66 (18)
C9A—C13A—C16A	122.08 (16)	O1B—C14B—H14C	110.2
C12A—C13A—C16A	121.23 (17)	C15B—C14B—H14C	110.2
O1A—C14A—C15A	107.55 (17)	O1B—C14B—H14D	110.2
O1A—C14A—H14A	110.2	C15B—C14B—H14D	110.2
C15A—C14A—H14A	110.2	H14C—C14B—H14D	108.5
O1A—C14A—H14B	110.2	F2B—C15B—F3B	105.5 (2)
C15A—C14A—H14B	110.2	F2B—C15B—F1B	107.3 (2)
H14A—C14A—H14B	108.5	F3B—C15B—F1B	107.3 (2)
F3A—C15A—F2A	105.6 (2)	F2B—C15B—C14B	113.3 (2)
F3A—C15A—F1A	107.3 (2)	F3B—C15B—C14B	113.3 (2)
F2A—C15A—F1A	106.2 (2)	F1B—C15B—C14B	109.8 (2)
F3A—C15A—C14A	113.8 (2)	C13B—C16B—H16D	109.5
F2A—C15A—C14A	113.79 (19)	C13B—C16B—H16E	109.5
F1A—C15A—C14A	109.6 (2)	H16D—C16B—H16E	109.5
C13A—C16A—H16A	109.5	C13B—C16B—H16F	109.5
C13A—C16A—H16B	109.5	H16D—C16B—H16F	109.5
H16A—C16A—H16B	109.5	H16E—C16B—H16F	109.5
C13A—C16A—H16C	109.5	HB1—OB—HB1'	19.7
H16A—C16A—H16C	109.5	HB1—OB—HB2	102.8
H16B—C16A—H16C	109.5	HB1'—OB—HB2	122.4
HA1—OA—HA2	109.7	HB1—OB'—HB1'	5.6
C7B—S1B—C8B	98.24 (9)	HB1—OB'—HB2'	102.8
C12B—O1B—C14B	116.91 (16)	HB1'—OB'—HB2'	104.6
C7B—N1B—C6B	104.74 (16)		
			
C7A—N2A—C1A—C2A	179.6 (2)	C7B—N2B—C1B—C2B	−179.7 (2)
C7A—N2A—C1A—C6A	0.33 (19)	C7B—N2B—C1B—C6B	−0.1 (2)
N2A—C1A—C2A—C3A	178.4 (2)	N2B—C1B—C2B—C3B	179.3 (2)
C6A—C1A—C2A—C3A	−2.4 (3)	C6B—C1B—C2B—C3B	−0.1 (3)
C1A—C2A—C3A—C4A	0.7 (3)	C1B—C2B—C3B—C4B	0.1 (4)
C2A—C3A—C4A—C5A	1.2 (4)	C2B—C3B—C4B—C5B	−0.2 (4)
C3A—C4A—C5A—C6A	−1.3 (3)	C3B—C4B—C5B—C6B	0.1 (4)
C4A—C5A—C6A—N1A	−178.5 (2)	C4B—C5B—C6B—N1B	−179.5 (2)
C4A—C5A—C6A—C1A	−0.3 (3)	C4B—C5B—C6B—C1B	−0.1 (3)
C7A—N1A—C6A—C5A	177.7 (2)	C7B—N1B—C6B—C5B	179.6 (2)
C7A—N1A—C6A—C1A	−0.6 (2)	C7B—N1B—C6B—C1B	0.1 (2)
N2A—C1A—C6A—C5A	−178.36 (18)	N2B—C1B—C6B—C5B	−179.5 (2)
C2A—C1A—C6A—C5A	2.2 (3)	C2B—C1B—C6B—C5B	0.1 (3)
N2A—C1A—C6A—N1A	0.2 (2)	N2B—C1B—C6B—N1B	0.0 (2)
C2A—C1A—C6A—N1A	−179.23 (17)	C2B—C1B—C6B—N1B	179.6 (2)
C6A—N1A—C7A—N2A	0.8 (2)	C6B—N1B—C7B—N2B	−0.2 (2)
C6A—N1A—C7A—S1A	−179.74 (14)	C6B—N1B—C7B—S1B	179.98 (16)
C1A—N2A—C7A—N1A	−0.8 (2)	C1B—N2B—C7B—N1B	0.2 (2)
C1A—N2A—C7A—S1A	179.75 (12)	C1B—N2B—C7B—S1B	−179.95 (15)
C8A—S1A—C7A—N1A	0.36 (18)	C8B—S1B—C7B—N1B	−0.7 (2)
C8A—S1A—C7A—N2A	179.76 (14)	C8B—S1B—C7B—N2B	179.57 (16)
C7A—S1A—C8A—C9A	178.84 (12)	C7B—S1B—C8B—C9B	179.89 (14)
C10A—N3A—C9A—C13A	−1.6 (3)	C10B—N3B—C9B—C13B	0.3 (3)
C10A—N3A—C9A—C8A	178.03 (17)	C10B—N3B—C9B—C8B	−179.8 (2)
S1A—C8A—C9A—N3A	0.16 (19)	S1B—C8B—C9B—N3B	−0.3 (2)
S1A—C8A—C9A—C13A	179.79 (13)	S1B—C8B—C9B—C13B	179.63 (16)
C9A—N3A—C10A—C11A	1.0 (3)	C9B—N3B—C10B—C11B	−0.5 (4)
N3A—C10A—C11A—C12A	0.4 (3)	N3B—C10B—C11B—C12B	0.1 (4)
C14A—O1A—C12A—C11A	−0.3 (3)	C14B—O1B—C12B—C11B	6.8 (3)
C14A—O1A—C12A—C13A	−179.93 (16)	C14B—O1B—C12B—C13B	−173.54 (19)
C10A—C11A—C12A—O1A	179.03 (18)	C10B—C11B—C12B—O1B	−179.9 (2)
C10A—C11A—C12A—C13A	−1.4 (3)	C10B—C11B—C12B—C13B	0.5 (3)
N3A—C9A—C13A—C12A	0.7 (3)	N3B—C9B—C13B—C12B	0.2 (3)
C8A—C9A—C13A—C12A	−178.93 (16)	C8B—C9B—C13B—C12B	−179.64 (18)
N3A—C9A—C13A—C16A	179.90 (17)	N3B—C9B—C13B—C16B	−178.9 (2)
C8A—C9A—C13A—C16A	0.3 (3)	C8B—C9B—C13B—C16B	1.2 (3)
O1A—C12A—C13A—C9A	−179.52 (15)	O1B—C12B—C13B—C9B	179.70 (18)
C11A—C12A—C13A—C9A	0.8 (3)	C11B—C12B—C13B—C9B	−0.7 (3)
O1A—C12A—C13A—C16A	1.2 (2)	O1B—C12B—C13B—C16B	−1.1 (3)
C11A—C12A—C13A—C16A	−178.39 (17)	C11B—C12B—C13B—C16B	178.5 (2)
C12A—O1A—C14A—C15A	−176.73 (17)	C12B—O1B—C14B—C15B	171.8 (2)
O1A—C14A—C15A—F3A	−60.9 (2)	O1B—C14B—C15B—F2B	−57.4 (3)
O1A—C14A—C15A—F2A	60.1 (2)	O1B—C14B—C15B—F3B	62.7 (3)
O1A—C14A—C15A—F1A	178.9 (2)	O1B—C14B—C15B—F1B	−177.4 (2)

### Hydrogen-bond geometry (Å, °)

**Table:**

*D*—H···*A*	*D*—H	H···*A*	*D*···*A*	*D*—H···*A*
N2A—H2AA···OA	0.86	1.95	2.771 (2)	161
OA—HA1···N1A^i^	0.83	2.00	2.806 (2)	161
N2B—H2BA···OB	0.86	1.98	2.799 (3)	160
OB—HB1···N1B^ii^	0.84	2.03	2.798 (3)	152

Symmetry codes: (i) *x*+1, *y*, *z*; (ii) *x*, *y*−1, *z*.
